# Paralysis of Various Organs, Due to Disease of the Heart

**Published:** 1873-03

**Authors:** 


### PARALYSIS OF VARIOUS ORGANS, DUE TO DISEASE OF THE HEART.

At the last meeting of the Sociétié Biologique de Paris, Mr. Joffroy, house-surgeon to La Pitié, presented a patient suffering from paralysis of various organs, due to disease of the heart. The patient's history is briefly this : He is seventeen years old. In March, this year, he suddenly lost the sight of one eye ; and a few days after he showed confusion of mind, difficulty of mastication, and almost complete hemiplegia. He was then taken to the hospital, and the following lesions were observed: complete paralysis of the lower part of the face; tightness of the jaws; almost complete immobility of the tongue, whence the difficulty of mastication, deglutition, and the articulation of words. The eye was affected with amblyopia, and on being examined with the microscope was seen to present considerable anæmia of the retina. Hemiplegia was very marked in the limbs. On examining the heart an intense bruit de soufflé, during the first sound, was discovered. This bruit disappeared and came according to the various attitudes which the patient was made to assume. Pulse and respiration normal. The various symptoms increased in intensity, and it was quite impossible for the patient to take food. Finally, by means of the œsophageal sound, food of various sorts was introduced into the stomach. The patient has, since that time, gained strength, and the symptoms have amended; contraction of the buccal muscles can be excited ; deglutition is now possible ; the tongue, however, is immobile, flat, and smooth, and suggests the idea of atrophy.

M. Joffroy thus explains the causation of the various symptoms just described. In the situation of the left auriculo-ventricular orifice there is a floating polypous production giving rise to the variable bruit de soufflé; embolism thrown into various parts of the brain ; through the vertebral arteries into the region which commands the movements of the mouth, tongue, and pharynx; through the arteries of the brain into the hemisphere opposite to the hemiplegic side; through the ophthalmic into the retina.—*London Lancet.*

